# Two Australian genome assemblies expand the genomic blueprint of giant kelp

**DOI:** 10.1186/s12864-026-13061-7

**Published:** 2026-06-12

**Authors:** Hugo J. Scharfenstein, Andrew Carroll, Jakop Schwoerbel, Cintia Iha, Rebecca Jordan, Anusuya Willis

**Affiliations:** 1 Australian National Algae Culture Collection, NCMI, CSIRO, Hobart, TAS Australia; 2 Google Inc., Mountain View, CA USA; 3 Environment, CSIRO, Sandy Bay, TAS Australia

**Keywords:** Comparative genomics, Genome assembly, Giant kelp, *Macrocystis pyrifera*

## Abstract

**Background:**

Giant kelp, *Macrocystis pyrifera* (order Laminariales), occurs across the temperate coasts of the Northern and Southern Hemispheres and is at high risk from ocean warming. Few giant kelp forests remain across the Southeast Australian shelf, and some sites are currently under active restoration. Genomic resources can greatly aid in the conservation of remnant populations and enhance restoration efforts. Reference genomes are a fundamental resource as they are either prerequisites for, or substantially improve, many analyses used in conservation genomics. A single reference genome is available for giant kelp, assembled from a Californian haploid specimen. However, increasing evidence of genetic divergence between Northern and Southern Hemisphere populations highlights the need for regionally representative reference genomes.

**Results:**

We present two genome assemblies from the vegetative tissue (diploid sporophyte) of Australian giant kelp specimens. We performed *de novo* genome assembly using long-read sequencing (PacBio HiFi and ONT R10.4 Simplex) and used the ONT reads for scaffolding, assembling 98–99% of the genomes into 35 pseudo-chromosomes. Genome sizes ranged from 528 to 534 Mbp, with BUSCO completeness of 96–97% and QV scores of 51–52. Functional annotation identified 17,330 − 17,832 genes in the Australian assemblies. Genomic divergence between Australian and Californian genomes was seven-fold greater than between Australian genomes (1.5% vs. 0.2%), supporting a northeast–southwest Pacific genetic divergence. Differences in gene ontology enrichment patterns were also observed between Australian and Californian genomes, reflected by differing patterns of enrichment in gene ontologies linked to energy metabolism, proteostasis and stress responses.

**Conclusions:**

These two new genome assemblies will serve as valuable resources for ongoing research into Australian giant kelp genetics, while providing the basis for genomics-guided conservation and restoration of remnant giant kelp forests in Australia.

**Supplementary Information:**

The online version contains supplementary material available at 10.1186/s12864-026-13061-7.

## Background

Kelp (order Laminariales) are large brown seaweeds that are key ecosystem engineers of coastal temperate reefs. They form underwater forests that provide fundamental ecosystem services [1], are harvested and cultured for food and other products [2] and are anchored in cultural practices [3]. *Macrocystis pyrifera*, also known as giant kelp, is the largest kelp species that can form towering structures over 30 m high. *Macrocystis pyrifera* is broadly distributed, spanning the North and South American Pacific coasts, New Zealand, Southeast Australia and the Subantarctic islands [4].

In the global geographical context, Australian and New Zealand populations are the most distant from the ancestral populations in the northeast Pacific [5]. In Australia, *M. pyrifera* is found along the coasts of the states of South Australia, Victoria and Tasmania. Over the last decades, giant kelp coverage has drastically declined throughout its Southeast Australian range [6]. This population collapse is particularly evident in East Tasmania, an ocean warming hotspot [7], where *M. pyrifera* cover has decreased by 95–98% since 1940 [8]. This decline is primarily attributed to the southward extension of the East Australian Current, bringing warmer, nutrient-poor waters to East Tasmania, especially to Northeast Tasmania [7]. This has exacerbated the frequency and severity of marine heatwaves in the area [9] and enabled the range expansion of the long-spined sea urchin *Centrostephanus rodgersii*, which prevents giant kelp recruitment and causes urchin barrens [10]. To reverse this drastic decline of this key habitat-forming species, extensive restoration of giant kelp forests is needed. Such efforts are now underway along East Tasmania [11] and beyond, such as on the Pacific coast of North America [12].

Genomic resources are increasingly incorporated into the conservation of threatened species, providing valuable information for their management to ensure preservation of genetic diversity or population structure [13–15]. The decreasing cost of high-throughput sequencing and democratisation of analysis software have increased the availability of genome-scale datasets, providing genomic perspectives of the genetic diversity and historical demography of threatened populations and species [16]. A reference genome (e.g., a highly contiguous, accurate, and annotated genome assembly) is fundamental to maximise use of high-throughput sequencing data, enabling read mapping and downstream genetic analyses. Additionally, high-quality reference genomes can aid in the development of molecular markers, which provide a rapid and cost-effective tool to screen individuals for traits of interest [17].

A single reference genome of *M. pyrifera* has been assembled from the haploid life stage (gametophyte) of a Californian specimen [18]. Access to a high-quality reference has spurred a wide range of insights into giant kelp biology, ranging from identifying genetic drivers of heat stress tolerance [19] and sexual differentiation at the developmental stage [20] to characterizing the species’ mutational landscape [21]. Notably, a recent population genomics study of giant kelp was able to leverage the Californian reference genome with whole genome sequencing data, revealing multiple genetic health risks in populations from Western Canada and Washington, USA [22], demonstrating the value of a reference genome and genetic data. Developing such genomic resources in Australia is urgently needed to inform the conservation of remnant giant kelp populations and enhance local restoration efforts.

While a fragmented draft assembly exists for a Peruvian *M. pyrifera* specimen [23], no scaffold-level, let alone chromosome-level, reference genome is currently available for giant kelp from the Southern Hemisphere, which limits population genetics studies to rely on the Californian genome. Recent studies have re-ignited the debate over the taxonomy of *M. pyrifera*, with reports of significant genetic divergence between Northern and Southern Hemisphere populations (F_ST_ = 0.71, d_XY_ = 0.0077) [5, 22], while northeast and southeast Pacific populations were estimated to have diverged around 14,000 years ago [24], leading to a proposal to recognise these populations as distinct species [25]. Given the apparent genetic divergence between Northern and Southern Hemisphere giant kelp, it is therefore important to use a Southern Hemisphere reference genome for genetic analyses, or risk producing misleading information, such as underestimating genetic diversity and population differentiation, distorting demographic histories and recombination landscapes [26, 27]. Creating a reference genome for Australia is also essential to inform local conservation and restoration efforts. Further reinforcing the need for a local reference genome is the notion that no single genome can account for all the genetic diversity within a species [28]. The availability of multiple reference genomes for a species can reduce errors in genomic characterisation [29], while providing the building blocks of a species’ pangenome.

To address this knowledge gap, we assembled the first giant kelp genomes from Australia to provide valuable resources to reduce bias inherent to relying on a single reference genome. We conducted long-read sequencing (PacBio HiFi and ONT R10.4 Simplex) to assemble the genomes of two Australian giant kelp individuals to pseudo-chromosomal scale. We compared genome structure and function between these two Australian genomes with the available reference from California.

## Methods

### Sample collection

**Fig. 1:**
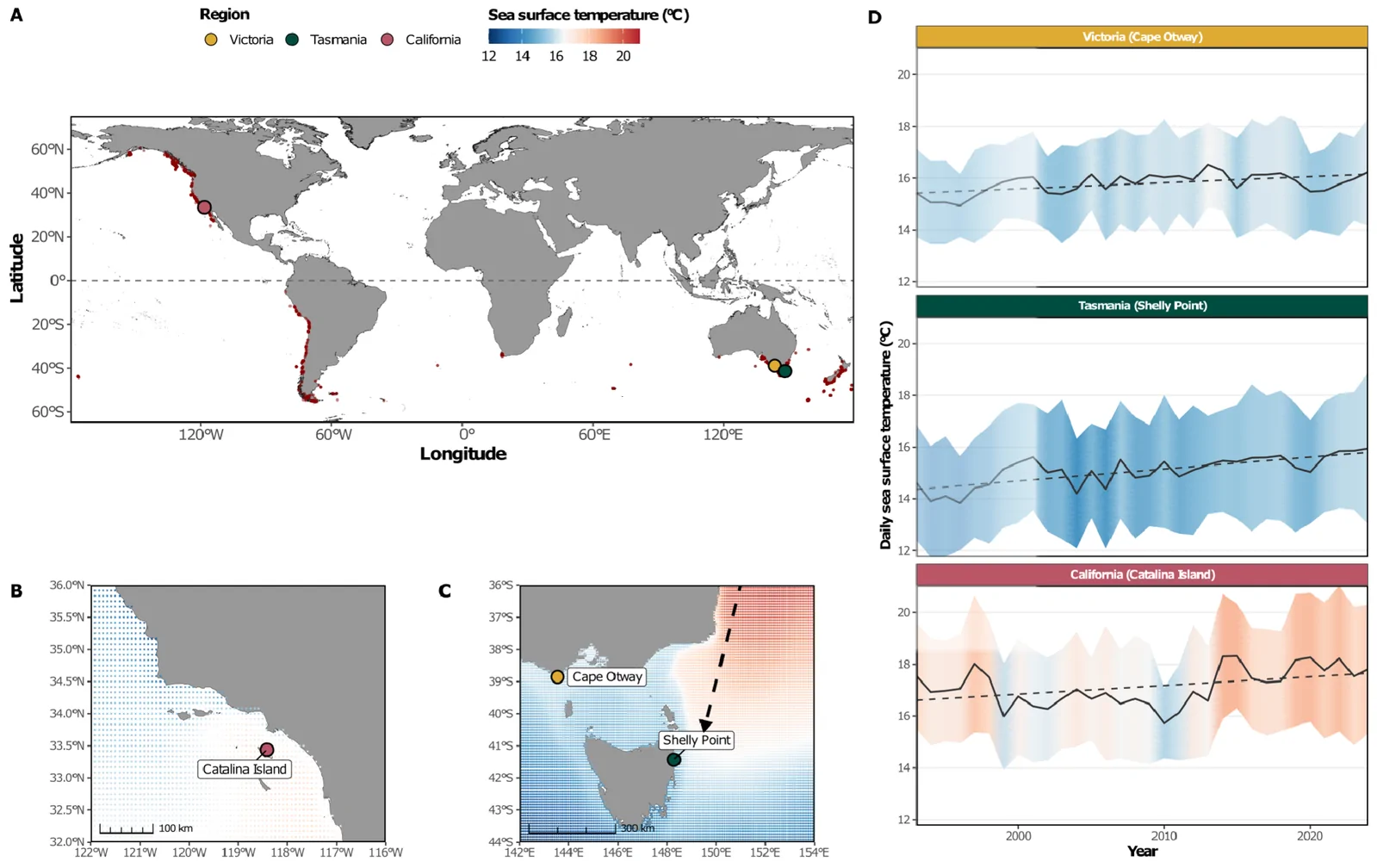
Geographic distribution of *Macrocystis pyrifera* and environmental context of genome assembly sampling sites. **A** Global distribution of *M. pyrifera* based on georeferenced occurrence records produced by Assis et al. [5], shown as small red points. Coloured circles indicate the sampling sites from which genome assemblies were generated: Cape Otway, Victoria; Shelly Point, Tasmania (this study); and Catalina Island, California (from Diesel et al.) [18]. The dashed horizontal line indicates the equator. **B**,** C** Climatological (1993–2024) mean sea surface temperature at the Californian (**B**) and Australian (**C**) sampling sites, derived from the Global Ocean Physics Reanalysis (GLORYS12V1) produced by Copernicus Marine Service. The dashed arrow indicates the approximate southward trajectory of the East Australian Current. **D** Annual mean sea surface temperature at each sampling site from 1993 to 2024 (solid line), with shaded ribbons indicating ± 1 standard deviation of daily values within each year. The dashed line shows the linear trend over the full time series

Two giant kelp individuals from the Southeast Australian Shelf were sampled in 2024, one at Crayfish Bay, Cape Otway, Victoria (sample ID: OTW_2, latitude: -38.8548, longitude: 143.5376) and the other around 600 km southeast at Shelly Point, Tasmania (SHL_1, -41.4353, 148.2785), under scientific research permit Nr. 23,090 (Department of Natural Resources and Environment Tasmania) (Fig. 1). Hereafter, the samples will be referred to by their region of origin (i.e., Victoria and Tasmania). For each individual, vegetative tissue from several apical blades was sampled, wiped with a 10% (v/v) bleach solution, rinsed under freshwater and blotted dry. The cleaned apical blades were snap frozen in liquid nitrogen and stored at -80 °C within 24 h of sampling.

### DNA extractions

High molecular weight DNA was extracted following a protocol modified from Diesel et al. [18]. (see Supplementary Information for the detailed DNA extraction protocol). Briefly, 70–90 mg (ww) of flash frozen giant kelp tissue was ground to a fine powder in a mortar topped up with liquid nitrogen. The powder was transferred to a CTAB lysis buffer, modified from Doyle & Doyle’s [30] original recipe, composed of: 1.2 M NaCl (CAS: 7647-14-5), 20 mM EDTA (CAS: 60-00-4), 100 mM Tris-HCl (CAS: 1185-53-1), 2% (w/v) CTAB (CAS: 57-09-0), 2% (w/v) PVP-40 (CAS: 9003-39-8), 1% (w/v) PEG 8,000 (CAS: 25322-68-3), 1% (v/v) β-Mercaptoethanol (CAS: 60-24-2) and 100 µg/ml Proteinase K (EO0492; Thermo Fisher Scientific). The ground tissue suspended in CTAB lysis buffer was incubated for 1 h at 55 °C, inverting every 15 min.

The lysate was centrifuged for 5 min and the supernatant mixed with an equal volume of Chloroform: Isoamyl Alcohol 24:1 (C0549, Sigma-Aldrich). The aqueous and organic phases were separated through centrifugation at 4 °C for 15 min. The upper aqueous phase was incubated for 30 min at 37 °C with RNAse A (12091021; Invitrogen) at a final concentration of 80 µg/ml. The lysate was mixed a second time with Chloroform: Isoamyl Alcohol and centrifuged at 4 °C for 15 min. The upper aqueous phase was mixed with 0.4x volume of 5 M Potassium Acetate (CAS: 127-08-2) and incubated for 20 min on ice. The lysate was centrifuged at 4 °C for 45 min. The resulting supernatant was mixed with 0.1x volume of 3 M Sodium Acetate (CAS: 127-09-3) and 0.8x volume of Isopropanol (CAS: 67-63-0). After a 30 min incubation at room temperature, the DNA was precipitated out of solution by centrifugation for 30 min. The pellet was washed twice with 70% Ethanol, air-dried for 3–5 min and resuspended in TE buffer. DNA quality control included an assessment of purity via NanoDrop spectrophotometry (thresholds: A_260/280_ between 1.8 and 2.0, A_260/230_ above 1.4) and quantitation using a Qubit dsDNA BR Assay Kit (ThermoFisher). Additionally, we used gel electrophoresis (0.4% agarose, 3 V/cm) to evaluate DNA size (GeneRuler High Range DNA Ladder; ThermoFisher) and check for signs of degradation (smearing).

### Sequencing

Two rounds of PacBio sequencing were carried out to overcome the relatively low output obtained from a single sequencing run. See Table S1 for a detailed breakdown of DNA input and sequencing output for each sample/run/sequence type. The samples were sequenced on the same PacBio Revio platform for both runs at the Biomolecular Resource Facility (Australian National University), Canberra, Australia. One round of ONT sequencing was carried out on a PromethION platform at the Biomolecular Resource Facility. ONT R10.4 reads were basecalled with *Dorado* v7.9.8 [31], using the ‘Super-accurate basecalling’ model at 400bps.

For each sample and sequencing run, 3.5–11.9 µg of DNA was sent for library preparation, which was carried out at the sequencing facility. Genomic DNA size was validated by Femto Pulse (Agilent), with a median fragment length of 25–50 Kbp (Table S1). Size selection was performed via Blue Pippin (Sage Science), with DNA recovery rates varying from 14 to 33% (Table S1). Samples were multiplexed on either one SMRT flow cell, for PacBio sequencing, or on one R10.4.1 flow cell, for ONT sequencing.

For ONT read correction and genome polishing purposes, both samples were sequenced on an Illumina NovaSeq 6000 platform (2 × 150 bp) by Azenta Life Sciences. For each sample, three replicate libraries were prepared. For each library, 200 ng of genomic DNA underwent library preparation at the sequencing facility using a VAHTS Universal Plus DNA Library Prep Kit for Illumina V2 (Vazyme), according to the manufacturer’s protocol. Each library was sequenced on a separate Illumina NovaSeq 6000 lane, with each sample multiplexed on the same lane.

### Sequencing read processing

HiFi reads were screened for contaminants using *fcs-gx* v0.5.5 [32] with the ‘--mask-transposons’ option turned off. HiFi reads were removed if they were identified as ‘EXCLUDE’ by *fcs-gx* (Table S2). Valid ONT R10.4 reads (i.e., minimum Q score of 10) were discarded if they were shorter than 10 Kbp (Table S2).

### Organellar genome assembly

Mitochondria and chloroplast genomes were assembled for each sample from the filtered HiFi reads using *oatk* v1.0 [33]. The resulting organellar genomes were annotated using *GeSeq* [34] and visualised with *OGDRAW* [35].

### Nuclear genome assembly

A summary of the software and settings used in the nuclear genome assembly and annotation pipelines is provided in Table S3, while an overview of the workflow is provided in Fig. S1. Primary and alternate assemblies were generated for both samples using *hifiasm* v0.25.0 [36] with filtered HiFi reads and ONT reads as input for scaffolding (using the --ul option).

The nuclear assemblies were then screened for the presence of organellar contigs. A BLAST search was carried out against a custom database composed of giant kelp organellar genomes assembled from the HiFi reads, as well as giant kelp organellar genomes retrieved from Iha et al. [37], Starko et al. [38], Chen et al. [39]. and GenBank (Accession ID: MW899036.1). In addition, the filtered HiFi reads were mapped back to the primary and alternate assemblies using *minimap2* v2.29 [40] to determine the coverage of each contig. A contig was removed if it presented a high similarity to the organellar genome database (i.e., an e-value lower than 1e-10 and qcov greater than 60%) and a high coverage (i.e., 3x greater than the sequencing coverage) or a GC content below 40%. The assemblies were then screened for contaminants using *blobtoolkit* v4.2.1 [41]. For each assembly, we obtained results from searches against the BLAST nucleotide database (v5, March 2025 release), UniProt Reference Proteomes database (April 2025 release) and BUSCO database (v5, March 2025 release). The results were added to *blobtoolkit* and contigs were filtered based on GC content (contigs with a GC content between 40 and 60% were kept) and taxonomy (contigs were kept if they were classified as Phaeophyceae, Bigyra or no-hit). See Figs. S2-S3 for blobplots.

The filtered assemblies underwent further duplicate purging using *purge_dups* v1.2.5 [42], with cutoffs for coverage thresholds set manually (Table S3). Cutoffs for *purge_dups* were determined for each assembly from coverage histograms. Low, mid and high coverage cutoffs were set to remove ‘junk’ contigs (contigs below low cutoff), test contigs for haplotypic duplication (contigs between low and mid cutoffs, the latter threshold corresponding to the transition between haploid and diploid coverages) and remove repetitive contigs (above high cutoff), respectively. The purged primary assemblies were scaffolded with ONT reads using *ntLink* v1.3.11 [43] in sensitive mode and with the minimum number of anchoring reads doubled (a = 2) for more stringent scaffolding. Gaps in the scaffolded assemblies were filled with *TGS-GapCloser* v1.2.1 [44] using ONT reads corrected with Illumina reads by *pilon* v1.24 [45].

The genome assemblies were polished using methods described by Nurk et al. [46]. Briefly, the PacBio HiFi reads were mapped to their own full assembly (i.e., primary, alternate, chloroplast, and mitochondrial assemblies). Variants were called with *DeepVariant* v1.9.0 [47] using the PacBio model. Single-nucleotide variants with a genotype quality of 20 or higher were used to correct the reference sequence. All assemblies underwent a final screening for contaminants using *fcs-gx* v0.5.5 before archiving on public databases (see Data Availability section for further detail).

### Genome annotation

The primary assemblies were soft masked sequentially using *RepeatMasker* v4.1.9 [48]. First, with Dfam’s database (release 3.9) [49], specifying Phaeophyceae for taxa search, and then with a *de novo* repeat library constructed from each primary assembly with *RepeatModeler* v2.0.2a [50]. Prior to second pass masking, gene fragments were removed from the *de novo* repeat library by conducting a BLAST search between the *de novo* repeats and a curated protein dataset, removing hits with *ProtExcluder* [51]. The protein dataset was built from the protein annotations of four Laminariales species, retrieved from the Joint Genome Institute’s Genome Portal: *M. pyrifera* (Portal ID: Macrocystis pyrifera CI_03 v1.0) [18], *Saccharina japonica* (Portal ID: Saccharina japonica str. Ja) [52], *Undaria pinnatifida* (Portal ID: Undaria pinnatifida M23) [53] and *Nereocystis luetkeana* (Portal ID: Nereocystis luetkeana NB12FB3 v1.0) [54]. Transposons were filtered from the combined protein dataset using *TransposonPSI* v1.0.0 [55].

Prediction of protein coding gene structures was carried out using *BRAKER* v3.0.7 [56], following the *BRAKER3* pipeline. As input for transcriptomic evidence, we retrieved 24 RNA-seq giant kelp datasets from NCBI’s Sequence Read Archive - BioProject accession IDs: PRJNA353611 (origin: Chile), PRJNA322132 (origin: Peru), PRJNA1116209 (origin: California, USA). As input for protein evidence, we combined the JGI protein datasets with two clade-partitioned (Stramenopiles and Viridiplantae) OrthoDB v12 [57] protein datasets. Transcriptomic and protein datasets then served as extrinsic evidence for training of *GeneMark-ETP* v1.02 [58] and *AUGUSTUS* v3.4.0, which also incorporated the output from *GeneMark-ETP*. The final gene set was predicted by *TSEBRA* v1.1.2.5 [59] with the combined results from *GeneMark-ETP* and *AUGUSTUS*. Functional annotation of the gene sets was performed using all available analyses from *InterProScan* v5.72-103.0 [60], as well as Phobius1.01 [61], SignalP v4.1 [62] and TMHMM v2.0 [63].

Gene prediction and functional annotation were also performed on the available masked Californian reference genome to provide a like-for-like comparison of gene content with the Australian genomes for orthology and gene ontology analyses.

### Assembly quality and genome features

Assembly contiguity was assessed via *assembly-stats* v1.0.1. Gene content completeness was evaluated via *BUSCO* (v5.8.0) [64] against the stramenopiles_odb10 (2024-01-08) dataset, using Augustus v3.5.0 [65] as gene predictor and with the flag ‘--long’. QV scores of the final assemblies were assessed with *yak* v0.1 [66].

*Circos* (v0.69.9) [67] plots of the primary assemblies were generated to depict: scaffold size, gene density over 1 Mbp bins, average GC content over 100 Kbp windows (using *bedtools* v2.31.1) [68], read coverage over 10 Kbp bins (generated with *minimap2* and *bedtools*), number of interspersed repeats (previously identified by *RepeatMasker*) over 100 Kbp bins and segmental duplications above 15 Kbp between contigs (identified with *asgart* v2.4.0) [69].

Male and female sex-determining regions (SDR) were identified via homology searches against the Victorian and Tasmanian genomes (primary and alternate assemblies). A female SDR protein set was generated from 21 female-restricted protein sequences identified by Liesner et al. [70], with a high homology to the Californian genome. The male SDR protein set comprised seven protein sequences identified as *M. pyrifera* orthologs of male SDR genes in *Ectocarpus sp* [71], including the Male INducer (*MIN*) gene reported by Luthringer et al. [72]. A BLAST search (tblastn) was carried out with the female/male SDR proteins sets against the Victorian and Tasmanian using *BLAST+* v2.11.0 [73]. Hits were kept if they presented on an e-value < 1e-10, qcov > 60% and pident > 40%. Filtered BLAST results were summarized for each assembly by quantifying the number of unique query proteins and total hits per target scaffold.

### Comparative genomics

Divergence between the Victorian, Tasmanian and Californian genomes was estimated using *Mash* v2.3 [74]. *Mash* divergence was also estimated between all three *M. pyrifera* genomes and three other Laminariales: *Saccharina japonica* [52], *Undaria pinnatifida* [53] and *Nereocystis luetkeana* [54]. We then performed a synteny analysis between all three *M. pyrifera* genomes using *ntSynt* v1.0.2 [75], which was visualised using *ntSynt-viz* v1.0 [76]. Using *OrthoFinder* v3.0.1b1 [77], we compared orthology between all three *M. pyrifera* genomes (Victorian, Tasmanian and California) and *Saccharina japonica* [52], *Undaria pinnatifida* [53] and *Nereocystis luetkeana* [54].

An enrichment analysis was carried out with the gene ontology (GO) terms previously identified by *InterProScan*. Prior to enrichment analysis, GO annotations were collapsed to the gene level by merging all gene isoform associated GO terms and removing duplicate GO terms. Pairwise comparisons were conducted between all three genome assemblies (Victoria, Tasmania, California). For each pairwise comparison, GO terms shared between both assemblies were identified. GO terms annotated with a greater number of genes in the focal assembly than in the comparison assembly were retained, forming the gene set of interest. The gene universe was constructed as the union of all annotated genes from both assemblies. GO enrichment analysis was then performed with *topGo* v2.58.0 [78], using the algorithm *weight01* and Fisher’s exact test to identify significantly enriched GO terms in each pairwise comparison. To focus on broader trends, a ‘nodeSize’ of 10 was used to prune the GO hierarchy from terms with less than 10 annotated genes. This approach identifies GO terms with an elevated gene annotation count in the focal assembly relative to the comparison, which may reflect differences in gene family size.

## Results

### Sequencing

PacBio Revio sequencing generated 3.5 M (44.7 Gbp) and 5 M (62.5 Gbp) HiFi reads for the Victorian and Tasmanian samples, respectively (Table S1). This corresponds to an 83x and 116x coverage of the 537 Mbp haploid *M. pyrifera* genome assembled by Diesel et al. [18]. A N50 of 14.4 Kbp was obtained across both samples and runs (Table S1). Contaminant filtering led to the removal of 64,604 (812 Mbp) and 4,980 (57 Mbp) HiFi reads from the Victorian and Tasmanian datasets.

ONT sequencing generated 0.55 M (12.15 Gbp) and 0.6 M (14.5 Gbp) R10.4 reads with a minimum Q score of 10 for Victorian and Tasmanian samples, respectively (Table S1). A N50 of 31 Kbp was obtained across both samples. Length filtering led to the removal of 0.16 M (0.68 Gbp) and 0.11 M (0.50 Gbp) ONT reads (Table S2).

### Organellar genomes

The mitochondrial genomes of the Victorian and Tasmanian samples are 37,313 and 37,297 bp long, respectively, with a GC content of 31.82% and 31.87% (Fig. S4). Both mitochondrial genomes contain 3 rRNA-coding genes (LSU rRNA, SSU rRNA, 5 S rRNA) and the same protein-coding genes (*n* = 37). The mitochondrial genomes differ in their tRNA content (30 for the Victorian genome vs. 28 for the Tasmanian genome). See Table S4 for a list of gene features in each mitochondrial genome.

The chloroplast genomes are 130,184 and 130,212 bp long for the Victorian and Tasmanian samples, respectively. GC content was at 30.88% for both samples (Fig. S5). The large single-copy region is 78,069 bp long for the Victorian genome, compared to 53,755 bp long for the Tasmanian genome, while the small single-copy region is 42,875 bp long for the Victorian genome (42,903 bp for the Tasmanian genome). Both chloroplast genomes share 3 rRNA-coding genes, 24 tRNA-coding genes and 144 protein-coding genes (Table S5).

### Nuclear genome assembly

*De novo* assembly generated primary assemblies at 557 Mbp (311 contigs) for the Victorian sample and 557 Mbp (549 contigs) for the Tasmanian sample, with corresponding N50 values of 7.1 Mbp and 7.4 Mbp (see Table S6 for assembly statistics throughout the assembly pipeline). The Tasmanian alternate assembly was three times the size (1.44 Gbp) of the Victorian alternate assembly (0.48 Gbp). Organellar contig and contaminant filtering led to the removal of 175 contigs (9.7 Mbp) and 330 contigs (19.6 Mbp) from the Victorian and Tasmanian primary assemblies, respectively (Table S6). Following duplicate purging, the Tasmanian alternate assembly was reduced to 523 Mbp (Table 1), comparable in size to the Victorian alternate assembly (473 Mbp).

**Fig. 2:**
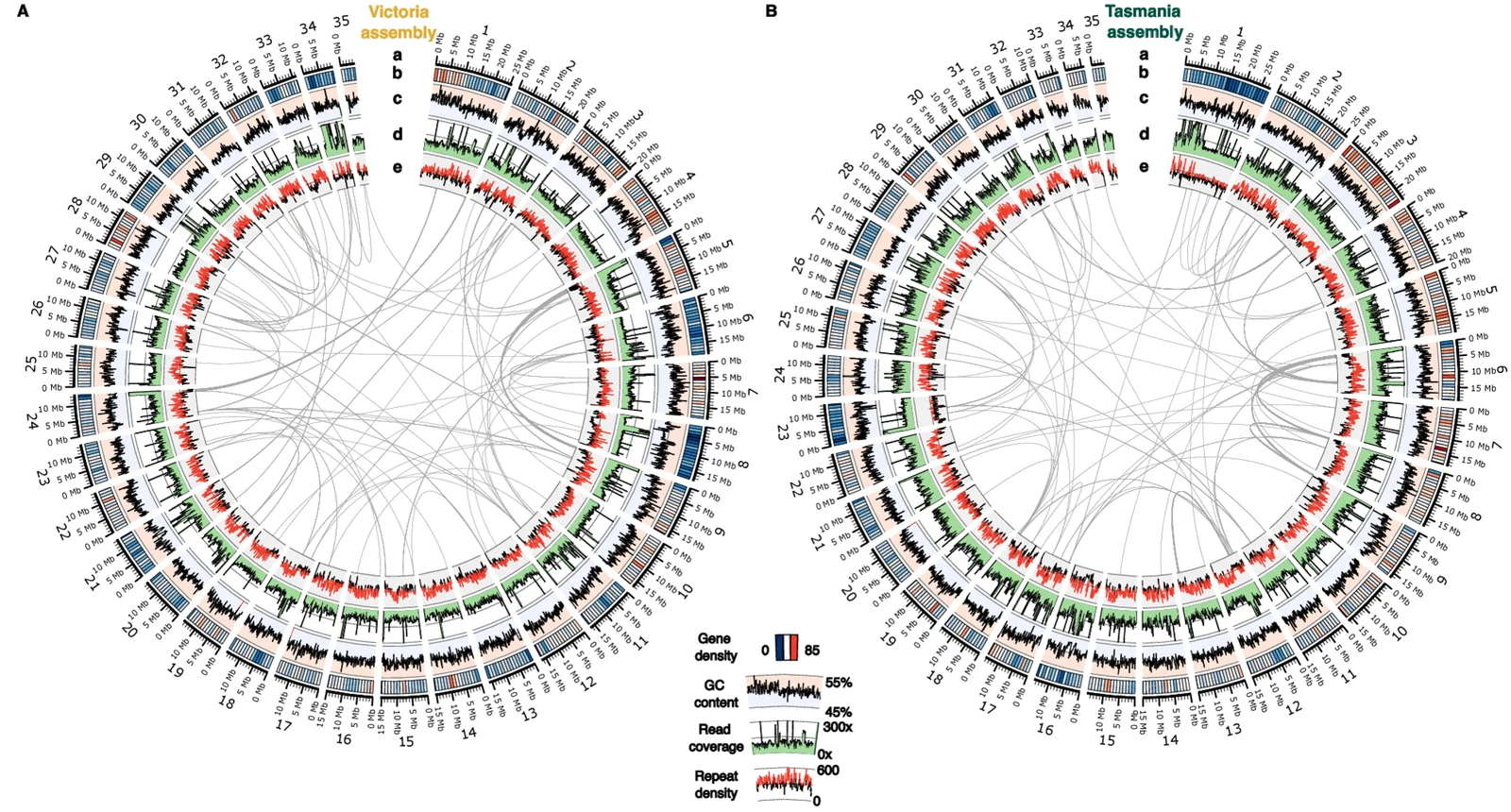
Genome features of the Victorian (**A**) and Tasmanian (**B**) primary assemblies. Characteristics of the 35 largest scaffolds (equivalent to 99.3% and 97.5% of the Victorian and Tasmanian assemblies, respectively) are shown in each concentric circle. (a) Scaffold size in Mbp. (b) Gene density over 1 Mbp windows (range: 0–85). (c) GC content over 100 Kbp sliding windows (range: 45–55%). (d) Read coverage over 10 Kbp sliding windows (range: 0-300x). (e) Number of interspersed repeats over 100 Kbp windows (range: 0-600). Inter-segment bands (links) represent segmental duplications between scaffolds greater than 15 Kbp

Scaffolding resulted in primary assemblies with 39 scaffolds/contigs (30 scaffolds, 9 contigs) and 49 scaffolds/contigs (23 scaffolds, 26 contigs) for the Victorian and Tasmanian genomes, respectively (Table 1). 99.3 and 97.5% of the Victorian and Tasmanian genomes assembled into 35 pseudo-chromosomes, respectively (Fig. 2). Polishing led to the correction of 1,419 and 2,671 single-nucleotide variants in the Victorian and Tasmanian assemblies, respectively.

**Table 1:** Comparison of summary statistics of the Victorian, Tasmanian and Californian giant kelp genome assemblies. For the Californian assembly, from Diesel et al. [18], the gene and gene isoform content metrics in parentheses were estimated in this study

Assembly type	Victoria		Tasmania		California
	Primary	Alternate	Primary	Alternate	
Assembly Size (Mbp)	534	473	528	523	537
Contigs	39	1,188	49	3,240	223
Largest contig (Mbp)	25.5	4.4	27.8	4.4	26.5
Contig N50 (Mbp)	15.2	0.9	15.0	0.3	13.7
Contig N90 (Mbp)	11.9	0.2	11.0	0.7	3.3
Gaps	61	0	47	0	698
N’s per 100 Kbp	0.1	0	0.1	0	13.0
Complete BUSCOS (C)	97	90	96	90	94
Single-copy BUSCOs (S)	97	90	96	90	94
Duplicated BUSCOs (D)	0	0	0	0	0
Fragmented BUSCOs (F)	0	2	1	6	1
Missing BUSCOs (M)	3	8	3	4	5
QV score	50.7	55.7	52.0	54.9	N/A
GC content (%)	50.2	50.0	50.2	50.0	50.4
Repeat content (%)	56.1	/	55.6	/	57.6
Genes	17,832	/	17,330	/	25,919 (18,400)
Gene isoforms	21,795	/	21,224	/	25,919 (21,418)

Final genome sizes of 534 and 528 Mbp were obtained for Victorian and Tasmanian primary assemblies. Gene content completeness, assessed via BUSCO analysis, was estimated at 97% and 96% for the Victorian and Tasmanian primary assemblies, respectively (Table 1). The N50 of both primary assemblies was assessed around 15 Mbp, with 47–61 gaps recorded. QV scores of 51 and 52 were obtained for the Victorian and Tasmanian primary assemblies, respectively.

### Genome features

GC content was estimated at 50.20% and 50.22% for the Victorian and Tasmanian assemblies, respectively, compared to 50.37% for the Californian reference (Table 1). Repetitive elements made up 56.1% and 55.6% of the Victorian and Tasmanian genomes, respectively, compared to 57.6% reported for the Californian reference [18]. Interspersed repeats represented the near totality (86–87%) of repetitive elements identified in the Australian genomes (Table S7). Gene prediction identified 17,832 genes (21,795 gene isoforms) and 17,330 genes (21,224 gene isoforms) for the Victorian and Tasmanian genomes, respectively. In comparison, 18,400 genes (21,418 gene isoforms) were predicted for the Californian genome.

All 21 female SDR-related proteins produced hits in the Victorian and Tasmanian primary assemblies (Table S8). In the Victorian genome, 16 female SDR proteins were localised to contig 31 (68 hits, mean pident = 77%, mean qcov = 91%), while scaffold 6 harboured eight female SDR proteins. Similarly, 16 female SDR proteins were localised to scaffold 1 in the Tasmanian genome (76 hits, mean pident = 75%, mean qcov = 92%). All seven male SDR proteins were identified in each genome. Scaffold 06 in the Victorian genome (50 hits, mean pident = 87%, mean qcov = 92%) and contig 27 in the Tasmanian genome (51 hits, mean pident = 86%, mean qcov = 92%) each harboured all seven male SDR proteins.

### Genome comparisons

Genome divergence between the Victorian and Tasmanian assemblies was estimated by *Mash* at 0.2% (912/1,000 matching hashes, *p* = 0.002). Both Australian genomes displayed a 1.5% *Mash* divergence with the Californian reference genome (566–568/1,000 matching hashes, *p* = 0.0153). *Mash* divergence estimates between all three *M. pyrifera* genomes and other Laminariales genomes increased with phylogenetic distance: 12% divergence with *Nereocystis luetkeana* (39–42/1,000 matching hashes, *p* < 0.001), 17–19% with *Saccharina japonica* (9–13/1,000 matching hashes, *p* < 0.001), and 20–21% with *Undaria pinnatifida* (6–7/1,000 matching hashes, *p* < 0.001).

**Fig. 3:**
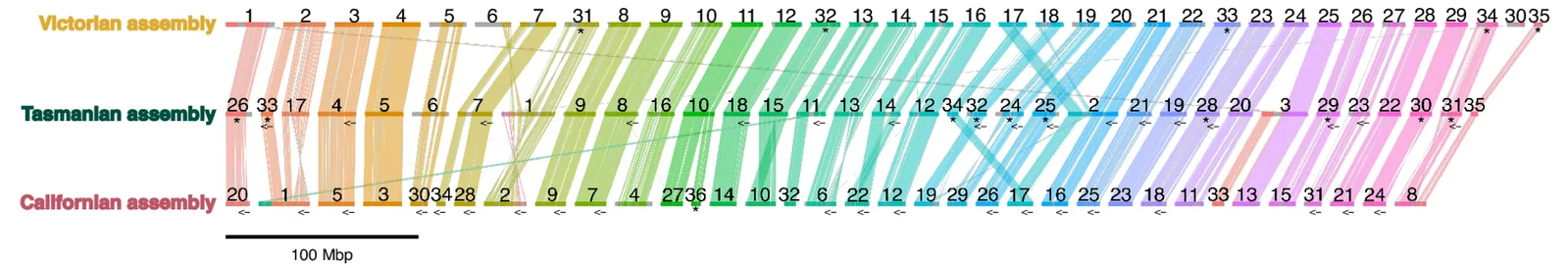
Cross-genome synteny between Australian and Californian genomes. Synteny blocks greater than 100 Kbp are depicted. For each genome, the 35 largest scaffolds/contigs are shown. Each scaffold/contig is labelled with its identifier from the relevant FASTA files. Arrows indicate reverse complementation of a synteny block. Asterisks indicate contigs. Each scaffold is represented by a different colour

In total, 94% (500 Mbp) of the Victorian genome was found to be in synteny with the Tasmanian genome, forming 1,137 synteny blocks with a N50 of 2.2 Mbp. In comparison, 86–87% (461 Mbp) of the Australian and Californian genome structures were in synteny. All three *M. pyrifera* assemblies displayed substantial genome-wide collinearity across their 35 largest scaffolds/contigs (Fig. 3). Rearrangements were observed between Australian genomes, notably at scaffold 17 of the Victorian assembly and at scaffolds 1 and 3 of the Tasmanian assembly. Scaffold 2 of the Victorian assembly was split in two in the Tasmanian assembly (scaffold 17 and contig 33), though not in the Californian reference (scaffold 1). Other structural differences between the Australian and Californian genomes include the splitting of scaffold 7 from both Australian assemblies into scaffolds 28 and 34 in the Californian assembly, and the splitting of scaffold 11/10 (Victorian/Tasmanian assembly, respectively) into scaffold 27 and contig 36 in the Californian assembly.

**Fig. 4:**
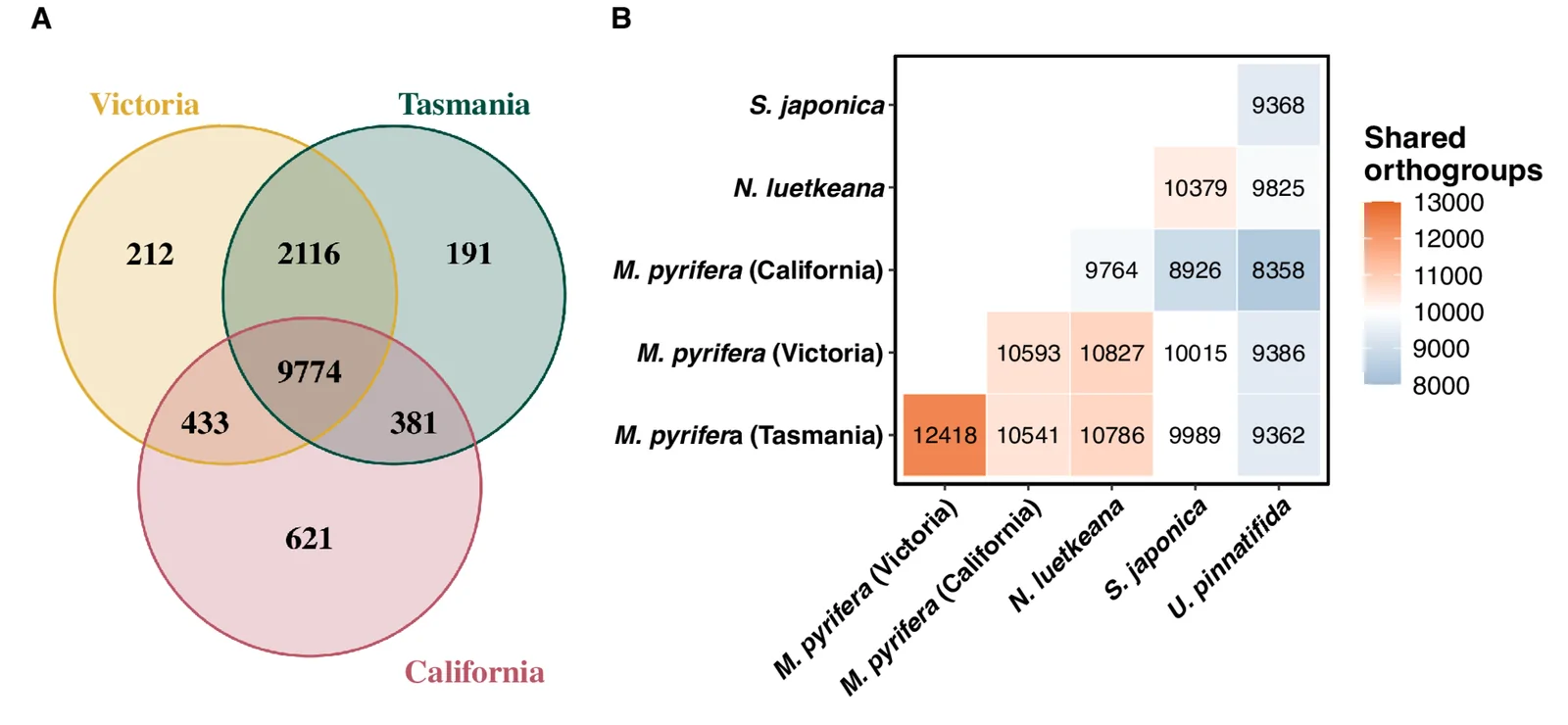
Orthology analysis between genome assemblies from four Laminariales species. **A** Venn diagram of shared orthologous groups between all three giant kelp genomes (Victorian, Tasmanian and Californian assemblies). **B** Shared orthologous groups between all three giant kelp genomes (*Macrocystis pyrifera*) and three other Laminariales species (*Nereocystis luetkeana*, *Saccharina japonica* and *Undaria pinnatifida*)

Orthology analysis identified 9,774 orthologous groups common to all three giant kelp genomes (Fig. 4A). Around five times more orthologous groups were shared between the Australian assemblies (2,116) than with the Californian assembly (381–433). Surprisingly, more orthologous groups were shared between the Australian giant kelp genomes and the Laminariales species *N. luetkeana* (10,786 − 10,827) than with the Californian genome (10,541 − 10,593) (Fig. 4B). All three giant kelp genomes shared the lowest number of orthologous groups with the *U. pinnatifida* genome.

**Fig. 5:**
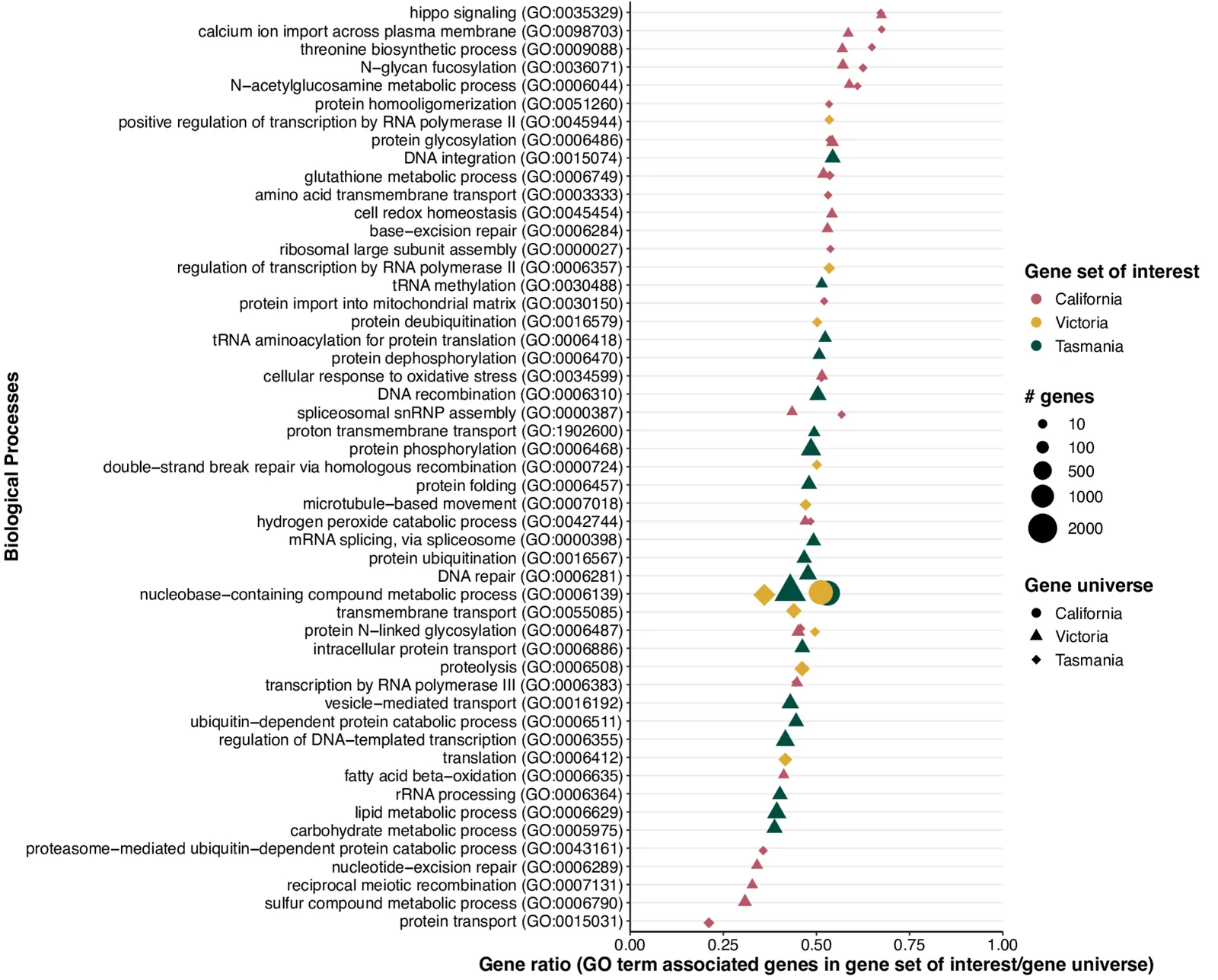
Comparative analysis of gene ontologies (GOs) enriched in Victorian, Tasmanian and Californian giant kelp genomes. For each pairwise comparison, the top 20 significantly enriched GO terms (p_Fisher_<0.01) from the category biological processes are shown here. GO terms pertaining to molecular functions and cellular components can be found in Fig. S6. A list of GO IDs can be found in Table S9. Significant differences were calculated with Fisher’s exact test

Relative to the counterpart Australian assembly, 34 gene ontology (GO) terms were significantly enriched (p_Fisher_<0.01) in the Victorian genome, compared to 53 GO terms in the Tasmanian genome (Table S9). Fewer GO terms (*n* = 12) were significantly enriched in the Australian genomes relative to the Californian genome than conversely (*n* = 111). Compared to the Victorian assembly, the Tasmanian genome showed a distinct enrichment in biological processes related to proteostasis (protein folding - GO:0006457- and protein ubiquitination - GO:0016567) and primary metabolism (ubiquitin-dependent protein catabolic processes - GO:0006511 -, lipid - GO:0006629 - and carbohydrate - GO:0005975 - metabolic process) (Fig. 5, Fig. S6). Conversely, the Victorian genome was enriched in biological processes linked to cellular homeostasis, such as protein deubiquitination (GO:0016579), proteolysis (GO:0006508), translation (GO:0006412) and transmembrane transport (GO:0055085). The Californian assembly was characterised by an enrichment in protein glycosylation (GO:0006487, GO:0006486), proteostasis (GO:0051085, GO:0044183) and oxidative stress response processes, including glutathione metabolic process (GO:0006749), glutathione transferase activity (GO:0004364), cell redox homeostasis (GO:0045454), cellular response to oxidative stress ( GO:0034599) and hydrogen peroxide catabolic process (GO:0006044).

## Discussion

We present the first genomes assembled from Australian *M. pyrifera* specimens using long read sequencing. Both assemblies are highly contiguous, at pseudo-chromosomal scale, and expand upon the current reference genome from California. They also represent the first genomes assembled from the sporophyte (diploid) life stage of giant kelp. Crucially, these two genomes can serve as the basis for future genetics studies of giant kelp in Australia, where the lack of population-scale genomic data has precluded a comprehensive understanding of population structure and adaptive variation. Together, the Victorian and Tasmanian assemblies presented here - spanning two ecologically distinct regions from the Australian range - provide a foundation for a future *M. pyrifera* pangenome. Such a resource is needed to move beyond the limitations of single individual reference genomes, capturing a wider breadth of genomic variation across the species’ global distribution - including locally adapted gene content that may differ substantially between Northern and Southern Hemisphere populations - and providing a more representative genomic blueprint for giant kelp research and conservation.

We assembled over 98% of each Australian genome into 35 pseudo-chromosomes, the same number of chromosome-level scaffolds as the Californian genome after Hi-C scaffolding by Diesel et al. [18]. In contrast, 92% of the Californian genome was assembled into 35 pseudo-chromosomes [18]. While the Australian and Californian genomes are highly contiguous, neither of the assemblies appears fully resolved at the chromosome-level. Historically, cytological studies have observed haploid giant kelp with 14–18 chromosomes [79–81] or 30–32 chromosomes [81, 82], with the large differences in chromosome number attributed to parthenogenesis. Cross-genome synteny revealed that two scaffolds in the Australian genomes are split in the Californian reference, while one scaffold in the Californian assembly is split in the Australian genomes. The *Undaria pinnatifida* genome, a closely related Laminariales species, was scaffolded into 30 chromosomes with Hi-C sequencing [53], further reinforcing the notion that all three giant kelp assemblies are insufficiently scaffolded to reach complete chromosome-level. While Hi-C scaffolding was attempted in this study, high optical duplications levels and low number of long-range chromatin interactions (> 20 Kbp), likely due to poor library preparation, precluded the use of Hi-C reads for chromosomal-level scaffolding. Optimisation of Hi-C library preparation protocols from giant kelp sporophyte tissue would benefit future attempts at generating *de novo* assemblies in giant kelp.

A key distinction between the Australian and Californian genomes is that they were generated from diploid sporophyte and haploid gametophyte samples, respectively. While haploid-derived assemblies typically offer higher contiguity due to a lack of heterozygosity [83], diploid assemblies present a unique insight into the dominant phase of giant kelp life history. Because sporophytes carry both U (female) and V (male) sex chromosomes, both sex-determining regions (SDRs) can be identified within a single diploid assembly. This stands in contrast to the Californian reference, which, being derived from a female gametophyte, contains only the female SDR. The female SDR in the Victorian and Tasmanian assemblies was localised to contig 31 and scaffold 1, respectively, both of which are in synteny with scaffold 2 of the Californian reference, previously identified by Diesel et al. as the location of a putative female SDR [18]. The male SDR was localised to scaffold 6 and contig 27 on the Victorian and Tasmanian genomes, respectively.

In the Tasmanian genome, we found the alternate assembly (i.e., the assembly representing the alternate haplotype) to be nearly three times the expected genome size, prior to purging (1.44 Gbp), and contained substantially more contigs than the Victorian alternate assembly (3,240 vs. 1,118). The nature of this larger assembly is unclear, but could be partly caused by highly heterozygous regions where haplotigs were insufficiently collapsed, leading to a more fragmented and inflated alternate assembly [42] - albeit mitigated by purging -, or be reflective of the presence of one or several additional haplotype(s), with polyploidy known to occur in the Laminariales [84–86]. Further research is needed to determine the karyotype of this individual and source population, which may explain the larger assembly.

Most genomic features were comparable between the Australian and Californian assemblies. The Australian genomes are similar in size to the Californian reference genome (528–534 Mbp for the Australian assemblies vs. 537 Mbp for the Californian assembly), providing further support that the haploid genome size is around 530–540 Mbp for giant kelp. Repetitive elements formed around 56% of the Australian giant kelp genomes, comparable to the Californian genome (57%) and other kelp genomes (e.g., 60% for *Saccharina sculpera*) [87].

Gene content was estimated at 17,330 − 17,832 genes for the Australian genomes. While considerably lower than the 25,919 genes identified by Diesel et al. [18], re-annotation of the Californian reference using *BRAKER3* identified 18,400 genes, a more comparable number of genes to the Australian assemblies. Hence, this discrepancy in gene content appears to be methodological, rather than biological. It may be attributed to the *BRAKER3* pipeline used here incorporating both protein and transcriptomic evidence, rather than solely transcriptomic evidence as was the case in Diesel et al. [18], and differences in gene prediction software used. Thus, by re-annotating the Californian reference, we likely minimised methodological confounding factors, ensuring subsequent orthology and gene ontology comparisons more accurately reflect biological variation, rather than technical variation. Nonetheless, artefactual variations in gene content likely persisted due to fundamental differences in assembly history between the Australian and Californian genomes. These disparities include varying gap content and scaffolding strategies, as well as the use of sequencing technologies with distinct accuracy (PacBio HiFi for the Australian assemblies vs. PacBio CLR for the Californian assembly).

Greater genomic divergence was observed between the Australian and Californian assemblies than between the Tasmanian and Victorian genomes (1.5% vs. 0.2%). The Australian genomes also displayed a higher degree of functional similarity, reflected by fewer significantly enriched GO terms relative to each assembly, compared to the Californian assembly. These findings are consistent with previous population genetics studies that have found genetic differences between Northern and Southern hemisphere giant kelp [5, 22, 24], highlighting the need for locally representative genome assemblies [29]. These findings raise the question of whether the northeast-southwest Pacific divergence observed here warrants the taxonomic rearrangement of *M. pyrifera* into multiple species. The 1.5% *Mash* divergence between Australian and Californian genomes is substantially smaller than the divergence between *M. pyrifera* and other Laminariales: 12% with *N. luetkeana*, 17–19% with *S. japonica*, and 20–21% with *U. pinnatifida*. This suggests that the northeast-southwest Pacific divergence within *M. pyrifera*, while substantial, remains below the divergence that separates recognised species within the order. Nevertheless, whether the genetic distance, in combination with the high inter-hemispheric divergence (F_ST_ = 0.71) [22] and potential structural genomic differences identified here, are sufficient to constitute species-level differentiation remains an open question. Resolving it will likely require broader population-level sampling and explicit tests of reproductive isolation.

Surprisingly, the Australian giant kelp genomes shared a slightly higher number of orthologous groups with the North American Pacific bull kelp (*N. luetkeana*) than with the Californian giant kelp genome. It should be noted that the *N. luetkeana* genome was annotated by the Joint Genome Institute using a similar pipeline to the original annotation for the California reference [18, 54], which yielded a higher gene count relative to the *BRAKER3*-based re-annotation performed here. Therefore, orthology patterns between *N. luetkeana* and the Australian giant kelp should be interpreted with caution, as shared orthologous groups may be inflated by methodological differences in genome annotation.

The Victorian and Tasmanian genomes showed differences in gene ontologies associated with primary metabolism, proteostasis and gene regulation. Relative to the Victorian genome, the Tasmanian genome was enriched in protein catabolism and lipid metabolic biological processes, which may indicate variation in energy acquisition, storage and membrane composition, as well as protein folding and ubiquitination processes consistent with enhanced proteostasis demands [88]. These differences may reflect the contrasting environmental conditions at each sampling site. Cape Otway, in South Victoria, lies at the eastern extent of the Bonney Coast upwelling system, which brings cooler, nutrient-rich water to the surface during the austral summer [89], while Shelly Point, in Northeast Tasmania, is exposed to rapid warming, nutrient-poor waters driven by the southward extension of the East Australian Current, which has increased marine heatwave frequency in the region [7]. The under-representation of translation and transmembrane transport processes in the Tasmanian genome may reflect adaptation to the strengthening of the East Australian Current, projecting warmer and nutrient-poorer water further southwards.

The Californian genome was enriched in oxidative stress and redox-related processes, including glutathione metabolism, a pathway central to mitigating reactive oxygen species generated during photosynthesis and environmental stress [90, 91]. This greater antioxidant capacity may reflect adaptation to a more variable or stressful environment relative to the Australian genomes. These differing enrichment patterns may reflect lineage-specific adaptive strategies to grow and survive in their respective environmental conditions. Further functional characterisation using gene transcript evidence is needed to determine whether the observed differences in GO term enrichments translate to functional differences between genomes.

## Conclusions

We provide two high-quality giant kelp genomes assembled to pseudo-chromosomal scale, which represent the first assemblies from the Australian distribution. We observed a high divergence between Australian and Californian genomes, both at the structural and gene ontology levels. Our findings reinforce the need for multiple, regionally representative genomes for the genomic characterisation of a species. These two high quality genomes from Australian specimens will aid in genomic evolutionary studies of this species and provide the foundation for genome-informed conservation of remnant giant kelp forests around the Southeast Australian shelf.

## Supplementary Information

Supplementary Material 1.

Supplementary Material 2.

## Data Availability

All data needed to evaluate the conclusions in this study are publicly available at CSIRO’s Data Access Portal (https://doi.org/10.25919/cfkr-fe18). The raw sequence files are deposited at NCBI’s Sequence Read Archive, under BioProject accession numbers PRJNA1405138 for OTW_2 and PRJNA1357414 for SHL_1. The primary and alternate genome assemblies generated in this study are archived at NCBI’s Genome database, under BioProject accession numbers PRJNA1405138 for OTW_2, and PRJNA1357414 for SHL_1. The primary assemblies archived at NCBI’s Genome database include their respective organellar genomes.Scripts needed to reproduce the genome assemblies and annotations are available on GitHub (https://github.com/hscharfenstein/Giant-kelp-genome-assemblies.git).
