# Supplementary material for: Two Australian genome assemblies expand the genomic blueprint of giant kelp
Source: BMC Genomics. 2026 Jun 12;27:697. doi: 10.1186/s12864-026-13061-7 (PMC13495250; doi:10.1186/s12864-026-13061-7)
Supplement: Supplementary file 1 [file 12864_2026_13061_MOESM2_ESM.docx]

**Supplementary Information**

**Supplementary Methods**

**DNA extraction protocol**

**Buffer preparation**

**1.** Set a water bath or oven to 55°C, used to dissolve the lysis buffer and during lysis.

**2.** Prepare the CTAB Lysis buffer fresh on the day of extraction.

CTAB Lysis buffer recipe (100 ml)

* Add the β-mercaptoethanol (10 µl per ml of lysis buffer) and Proteinase K (5 µl per ml of lysis buffer) prior to starting Step 6.

**3.** Incubate at 55°C and mix the lysis buffer for at least 15 min. Ensure the solution is clear before use. Keep at 55°C until ready to start Step 5.

**Cell lysis**

**4.** Dispense 1.5 ml of CTAB Lysis buffer to a 2 ml Lysis tube. If performing a large prep, then dispense 30 ml of CTAB Lysis buffer to a 50 ml Falcon tube.

**5.** Add the ground giant kelp tissue to the CTAB lysis buffer.

**6.** Incubate the samples at 55°C for 1 hour, shaking at 300 rpm if possible. Invert every 10-15 min.

**7.** Cool tubes for 5 min at RT then centrifuge at 3,000 g for 5 min at room temperature (RT). For large preps, centrifuge at 1,000 g.

**8.** Transfer 1 ml of lysate to a new 2 ml Protein LoBind tube. Avoid carryover of any debris. For large preps, transfer 20 ml of lysate to a new 50 ml Falcon tube.

**Contaminant removal**

**9.** Add an equal volume (1 ml) of Chloroform: Isoamyl (24:1). For large preps, add 20 ml of Chloroform: Isoamyl (24:1).

**10.** Mix by inverting 10-15 times. Let stand at RT for 2-3 min. Centrifuge at 10,000 g for 15 min at 4°C. For large preps, centrifuge at 3,000 g.

**11.** Transfer to a new tube the aqueous phase (top layer), which should be ~900 µl (~15 ml for large preps). Avoid carryover of the organic phase.

**12.** Add 4 µl of RNase A (final concentration: 80 µg/ml). Incubate at 37°C for 30 min. For large preps, add 40 µl of RNase A.

**13.** Re-extract the aqueous phase with an equal volume (900 µl) of Chloroform: Isoamyl (24:1). For large preps, add 15 ml of Chloroform: Isoamyl (24:1).

**14**. Centrifuge at 10,000 g for 15 min at 4°C. For large preps, centrifuge at 3,000 g.

**15.** Transfer 750 µl of supernatant to a 1.5 ml DNA LoBind tube (the supernatant is typically ~900 µl). For large preps, transfer 12.5 ml to a 15 ml Falcon tube.

**16.** Add 0.4 x volume of the supernatant (~300 µl) of 5 M Potassium Acetate. For large preps, add 5 ml of 5 M Potassium Acetate.

**17.** Invert 10 times. Incubate on ice for 20 min.

**18.** Centrifuge at 3,500 g for 45 min at 4°C. Decant ~1 ml of supernatant to a new 1.5 ml DNA LoBind tube. For large preps, decant ~17.5 ml to a 50 ml Falcon tube.

**DNA precipitation and washes**

**19.** Add 0.1 x volume of the supernatant (100 µl) of 3 M Sodium Acetate. For large preps, add 1.75 ml of 3 M Sodium Acetate.

**20.** Add approximately 0.7 x volume of Isopropanol (770 µl). Mix gently, invert 10 times. For large preps, add 13.5 ml of Isopropanol.

**21.** Incubate at RT for 30 min.

**22.** Centrifuge at 10,000 g for 30 min at RT. For large preps, centrifuge at 3,000 g.

**23.** Discard the supernatant. Add 750 µl of 70% Ethanol. Gently mix by inversion. For large preps, add ~10 ml of 70% Ethanol (enough to cover the pellet).

**24.** Centrifuge at 10,000 g for 10 min at RT. For large preps, centrifuge at 3,000 g.

**25.** Remove supernatant.

**26.** Repeat Steps 23-25 once more (i.e., two washes in total).

**27.** Let the pellet dry 3-5 min.

**28.** Resuspend in 50 µl of TE buffer. For large preps, resuspend in 100-200 µl, pending on pellet size.

**29**. Let resuspend overnight.

**DNA cleanup**

The extracted DNA needs to be further cleaned before long-read sequencing as contaminants (primarily polysaccharides) are likely still present after extraction.

**30**. Aliquot 10-30 μg of DNA into a 1.5 mL DNA LoBind tube. Increase the volume to 200 μL with 10 mM Tris-HCl (pH 8).

Note: Volume can exceed 200 μl, maximum of 600 μl due to tube capacity at later steps (ethanol precipitation).

**31.** Add RNAse A (1 µl) and Proteinase K (1 µl) to the solution.

Note: Target concentrations of each enzyme is 100 μg/ml.

**32.** Incubate the samples at 55°C for 20 min, shaking at 400-500 rpm.

**33.** Increase the volume to 600 μl with 10 mM Tris-HCl pH 8 (add 400 μl).

**34.** Add an equal volume of Chloroform: Isoamyl (24:1) (600 μl). Mix by inverting 10-15 times.

**35.** Centrifuge at 10,000 g for 1 min at RT.

**36.** Transfer the upper aqueous phase to a new 1.5 mL DNA LoBind tube.

**37.** Repeat the Chloroform: Isoamyl (24:1) cleanup. Supernatant should be ~600 μl.

**38.** Add 1.5 x volume of 100% Ethanol (~900 μl) and 0.1x volume of 3 M Sodium Acetate (~60 μl).

**39.** Incubate on ice for 1 min.

**40.** Centrifuge at 10,000 g for 1 min at 20°C (or RT).

Note: HMW DNA should pellet easily. If no pellet can be seen, centrifuge for longer. Bringing Ethanol concentration to 2x volume is also an option.

**41.** Carefully decant the supernatant as soon as possible, without disturbing the pellet.

**42.** Add approximately 700 µl of freshly prepared 70% Ethanol.

**43.** Let the pellet soak for 1 min at RT to dissolve excess salts.

**44.** Centrifuge at 10,000 g for 1 min at RT.

**45.** Carefully decant the supernatant as soon as possible, without disturbing the pellet.

**46.** Repeat Steps 42-45 for a second 70% Ethanol wash

**47.** Air-dry the pellet for 5 min.

**48.** Dissolve DNA with 100 µL of 10 mM Tris-HCl (pH 8) or TE buffer.

**Supplementary Figures**

**Figure S1: Overview of the genome assembly pipeline for the Victorian and Tasmanian assemblies.**

**Figure S2: Blobplots of the primary (A) and alternate (B) genome assemblies for the Victorian sample (OTW).**

**Figure S3: Blobplots of the primary (A) and alternate (B) genome assemblies for the Tasmanian sample (SHL).**

**Figure S4: Mitochondrial genomes from Victorian (A) and Tasmanian (B) samples.**

**Figure S5: Chloroplast genomes from Victorian (A) and Tasmanian (B) samples.**

**Figure S6: Enriched gene ontologies (GOs) enriched in Victorian, Tasmanian and Californian giant kelp genomes**. The top 20 significantly enriched GO terms (p<0.01) for each pairwise comparison are shown here.
